# An evaluation of the short physical performance battery following pulmonary rehabilitation in patients with chronic obstructive pulmonary disease

**DOI:** 10.1186/s13104-018-3458-7

**Published:** 2018-06-04

**Authors:** Petra Larsson, Christine Råheim Borge, Malin Nygren-Bonnier, Anners Lerdal, Anne Edvardsen

**Affiliations:** 1 Section of Physiotherapy, Department of Surgery, Lovisenberg Diaconal Hospital, Postboks 4970, 0440 Oslo, Norway; 2 Department of Research, Lovisenberg Diaconal Hospital, Postboks 4970, 0440 Oslo, Norway; 3 Department of Health Sciences, Faculty of Medicine, Institute of Health and Society, University of Oslo, Blindern 0317, Postboks 1089, Oslo, Norway; 4 Division of Physiotherapy, Department of Neurobiology, Care Sciences and Society, Karolinska Institutet and Functional Area Occupational therapy and Physiotherapy, Allied Health Professionals Function, Karolinska University Hospital, Huddinge, Karolinska Institutet, 171 77 Stockholm, Sweden; 5 Department of Nursing Science, Faculty of Medicine, Institute of Health and Society, University of Oslo, Blindern 0318, P.O. Box 1130, Oslo, Norway; 6 Department of Respiratory Physiology and Exercise Physiology, LHL Hospital Gardermoen, Ragnar Strøms veg 10, 2067 Jessheim, Norway

**Keywords:** Exercise test, Pulmonary disease, Chronic obstructive, Rehabilitation

## Abstract

**Objective:**

There is a need for simple tools to evaluate physical performance in patients with COPD before and after pulmonary rehabilitation. The aims of this study were to evaluate changes in short physical performance battery (SPPB)-scores in patients with COPD after a 4-week pulmonary rehabilitation program; explore possible relationships between SPPB-scores and exercise capacity (6-min walk distance), dyspnea (modified Medical Research Council’s dyspnea scale), disease-specific quality of life (COPD assessment test), and pulmonary function (predicted forced expiratory volume in one second) at baseline; and explore if changes in SPPB-scores are related to changes in exercise capacity, dyspnea, and disease-specific quality of life following pulmonary rehabilitation.

**Results:**

Forty-five patients with COPD were included in the final analysis. SPPB-scores improved following pulmonary rehabilitation (mean change: 1.2 ± 1.7 points, p < 0.001). There were moderate correlations between SPPB-scores and exercise capacity (r = 0.50, p < 0.001) and dyspnea (r = − 0.45, p = 0.003) at baseline, but not with pulmonary function or disease-specific quality of life. Changes in SPPB-scores were not associated with changes in exercise capacity or dyspnea scores. The SPPB may be a useful tool for evaluating physical performance in COPD

*Trial registration* ClinicalTrials.gov NCT02314338, December 11, 2014.

## Introduction

Patients with chronic obstructive pulmonary disease (COPD) often experience respiratory related symptoms [1] which can lead to functional limitations [2, 3], influence quality of life and mortality [1], and are associated with increased longitudinal risk of disability [4]. The severity of COPD can be classified in GOLD stages I–IV based on airway obstruction, where I is mild, II is moderate, III is severe, and IV is very severe [1]. However, physical performance is reported to be more useful for prognosis than airway obstruction [5]. One aim of pulmonary rehabilitation (PR) is to reduce functional limitations by improving physical performance. Therefore, valid and reliable physical performance tests are needed.

The most commonly used physical performance tests in COPD are field walking tests [6]. However, they require substantial time, space, and equipment, making them impractical in many settings. The Short Physical Performance Battery (SPPB) is a simple test of lower extremity function [7, 8]. It is comprised of three subtests; standing balance, four-meter gait speed (4MGS), and five sit-to-stand (5STS). The subtests are scored from 0 to 4 and summarized into the SPPB score (range 0–12 points), with higher scores reflecting better performance. Traditionally, the SPPB is used as a screening tool to identify older adults who may benefit from interventions aimed at delaying or preventing age-related disability [8]. Because the SPPB can be administered in a variety of different settings (e.g. private homes, in- and out-patient wards, nursing homes), it can be used instead of, or in addition to, field walking tests for evaluating physical performance before and after interventions in patients with COPD. To our knowledge, there are no studies investigating whether SPPB scores changes following PR in patients with COPD. Furthermore, few studies have investigated if exercise tolerance, dyspnea, disease-specific quality of life (DSQoL), and pulmonary function correlate with SPPB scores in patients with COPD [9, 10]. Thus, the aims of the present study were to: (a) evaluate changes in SPPB scores among patients with COPD during a 4-week, PR-program, (b) explore possible relationships between the SPPB scores and exercise capacity, dyspnea, DSQoL, and pulmonary function at baseline, and (c) explore whether changes in SPPB scores are related to changes in exercise capacity, dyspnea, and DSQoL during PR.

## Main text

### Methods

**Fig. 1:**
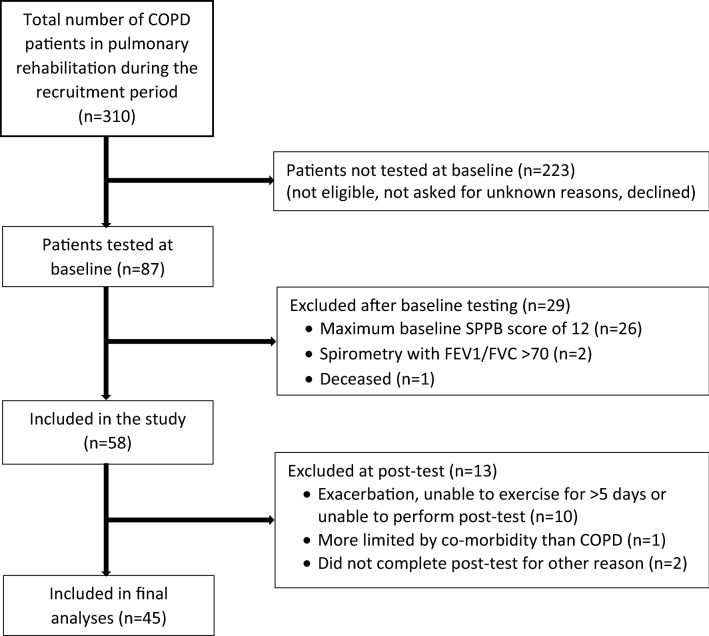
Flow-chart of patient selection. *COPD* chronic obstructive pulmonary disease, *SPPB* short physical performance battery, *FEV1/FVC*: forced expiratory volume in one second/forced vital capacity

This quasi-experimental study included a sample of consecutively recruited patients enrolled in a four-week, in-patient PR-program at LHL-Clinics, Glittre, Norway. This site were chosen for practical reasons (i.e. likelihood of reaching recruitment goals). The recruitment period was January to June 2015. Forty-five patients were included in the final analysis (Fig. 1). Inclusion criteria were a diagnosis of COPD, cognitive ability to provide informed written consent, and ability to understand and complete questionnaires. Exclusion criteria were ongoing exacerbation of COPD, inability to exercise, and co-morbidities limiting the patient’s physical performance more than the COPD alone (e.g., neurological disorder, severe angina). To minimize the ceiling effect, patients considered likely to achieve the maximum score of 12 on the SPPB were excluded prior to baseline testing. Exclusion on this ground, was determined by which test protocol that was used on the progressive treadmill test the patients was tested with in the beginning of PR at LHL-clinics Glittre. All patients tested with protocol 4 were excluded, because the initial walking speed (4.8 km/h) suggested that the patients would have scored 12 points (maximum) on the SPPB. Patients who obtained the maximum SPPB score at baseline and patients who did not exercise for five consecutive days or more prior to the post-test were excluded.

### Outcome measures

Background data, including GOLD stages, were obtained from patients and medical records at baseline. All tests were administered by qualified health-care professionals. Exercise habits during PR were reported in a training log.

### Primary outcome measure

The SPPB is comprised of three subtests: a hierarchical standing balance test (feet placed side-by-side, semi-tandem, and tandem, for 10 s each); a 4MGS test (timed four-meter walk test at habitual gait speed); and a 5STS test (timed five-repetition chair stands test performed as fast as possible). For scoring, see Background. The SPPB is reliable [11] and valid [7]. A one-point change is considered clinically meaningful [7]. Standardized instructions were followed, but in light of a possible learning effect [12], the SPPB was performed twice at baseline and twice at the end of PR. The patients rested for a minimum of 5 min before taking the test again. The best score was used in the data analysis.

### Secondary outcome measures

The secondary outcome measures were administered at baseline and at the end of PR except for pulmonary function, which was measured at baseline only.

#### Exercise capacity

Exercise capacity was measured as distance walked (6MWD) during the six-minute walk test (6MWT). It is frequently used for measuring response to therapeutic interventions in COPD [13]. The 6MWT was performed according to the standardized protocol [6].

#### Dyspnea

The impact of dyspnea on daily activities was measured with the modified Medical Research Council dyspnea scale (referred to as the mMRC) [14]. The mMRC consists of five grades (range 0–4), where a higher number represents a higher degree of breathlessness on daily activities [15]. The mMRC is valid for assessing dyspnea [16].

#### Disease-specific quality of life

Disease-specific quality of life was measured using the COPD Assessment Test (CAT) [17]. The CAT consists of eight items designed to assess the impact of COPD on health status (range 0–40) and is a valid and reliable measure [17].

#### Pulmonary function

Pulmonary function (i.e. predicted forced expiratory volume in one second (FEV1%) was measured by spirometry (MasterScreen PTF, Jaeger GmbH, Würtzburg) in accordance with ATS/ERS guidelines [18].

### Pulmonary rehabilitation

The PR-program consisted of individual and group-based strength and endurance training, education, and individual sessions with a multi-professional health-care team.

### Statistics

Data analyses were performed using IBM SPSS Statistics version 22.0 for Windows (SPSS Inc., Chicago, IL, USA). Descriptive data were presented as mean and SD, or number and percentage out of total sample. Paired Student’s *t* test were used to evaluate pre- and post-test changes at group level. Results were confirmed with non-parametric statistics when the criterion of a normal distribution was not met. Analyses were performed per-protocol and confirmed with intention-to-treat. Cohen’s d was calculated to estimate the effect size of changes in SPPB scores with 0.2 indicating a small effect size, 0.5 a medium effect size, and 0.8 a large effect size [19]. Effect size was calculated using an online calculator [20, 21]. Sample size calculation for the main outcome measure was based on 80% power to detect a one-point change in SPPB score (SD 2.5), with an alpha level of 0.05. The estimated sample size was forty. Relationships between the SPPB and 6MWD, mMRC, CAT, and FEV1% were assessed with Pearson’s correlation coefficient. To explore relationships between changes in SPPB scores and changes in 6MWD and mMRC scores, two separate multiple regression analyses were performed. The significance level was set at p < 0.05.

## Results

Forty-five patients in GOLD stages II-IV were included in the final analysis (Fig. 1) Baseline characteristics are shown in Table 1.

**Table 1:** Patient characteristics at baseline (n = 45)

	N (% of total sample) or Mean ± SD	Range
Sex		
Male	20 (44)	
Female	25 (56)	
Age, years	69 ± 6	58–85
Height, cm	168.5 ± 10.3	146–188
Weight, kg	70.5 ± 20.1	38.9–114.9
BMI, kg/m^2^	24.9 ± 7.1	14.6–44.4
FEV1%, % of predicted	42.1 ± 13.2	21.0–74.0
FEV1/FVC, %	43.6 ± 9.5	26.7–66.3
GOLD classification		
GOLD II	13 (29)	
GOLD III	22 (49)	
GOLD IV	10 (22)	
Comorbidities (including pulmonary)	3.5 ± 1.7	1.0–9.0
Current smoker	10 (22)	
Smoking pack years (n = 35)	43 ± 18	20–120
Exercise frequency		
< 3 times per month	11 (24)	
2–4 times per month	3 (7)	
Once a week	9 (20)	
2–3 times per week	18 (40)	
> 3 times per week	4 (9)	
6MWD in meters	388 ± 99	190–535
CAT (n = 42)	22 ± 6	10–34

Data are presented as n (% of total sample) or mean ± SD (range)

*BMI* body mass index, *FEV1%* forced expiratory volume in one second as percent of predicted value, *FEV1/FVC* forced expiratory volume in one second/forced vital capacity, *GOLD* global initiative for chronic obstructive lung disease, *6MWD* six-minute walk distance, *CAT* COPD assessment test

All included patients reported to have exercised at least two to three times per week for the duration of the PR program.

The mean increase in SPPB score was 1.2 ± 0.9 points, p < 0.001 (Table 2). Intention-to-treat analysis (n = 59) did not change the results.

**Table 2:** Mean SPPB, 6MWD and mMRC score at baseline (pre-test) and after 4 weeks of pulmonary rehabilitation (post-test)

	n	Pre-test	Post-test	Change	Effect size	p value
SPPB total score	45	9.9 ± 1.7	11.1 ± 1.7	1.2 ± 0.9	0.7	< 0.001
SPPB subtests						
Balance	45	3.7 ± 0.7	3.8 ± 0.7	0.0 ± 0.4	0.0	0.735
4MGS	45	3.5 ± 0.7	3.8 ± 0.4	0.3 ± 0.5	0.5	< 0.001
5STS	45	2.6 ± 1.0	3.5 ± 0.9	0.9 ± 0.7	0.9	< 0.001
6MWD	35	410 ± 88	426 ± 99	16 ± 53	0.2	0.071
mMRC	42	2.8 ± 0.9	2.2 ± 1.2	-0.6 ± 1.2	0.5	0.003

Data are presented as mean ± SD and Cohen’s d effect size of change

*SPPB* short physical performance battery, *4MGS* four-meter gait speed, *5STS* five sit-to-stand, *6MWD* six-minute walk distance, *mMRC dyspnea scale* modified Medical Research Council dyspnea scale

There was no change in the SPPB balance subtest, but the 4MGS and 5STS improved significantly with PR, with mean increases of 0.3 ± 0.5 points and 0.9 ± 0.7 points, respectively, both p < 0.001 (Table 2). At baseline, higher SPPB scores correlated with better performance on the 6MWD (r = 0.50, p < 0.001) and lower mMRC scores (r = − 0.45, p = 0.003). SPPB scores were not correlated with FEV1% or CAT at baseline. Multiple regression analyses indicated no associations between changes in SPPB scores and changes in either 6MWD (B = 0.001, p = 0.754) or mMRC scores (B = 0.113, p = 0.374). Changes in CAT scores were not analyzed because of missing data. CAT is part of routine testing at the LHL-clinics Glittre. However, for unknown reasons post-test data on CAT scores were missing from the medical records in 23 patients. Changes in mMRC scores and 6MWD are shown in Table 2.

## Discussion

The main findings of this study was that SPPB scores, and subtest scores of 4MGS and 5STS, improved significantly with PR in patients with COPD. Furthermore, higher SPPB scores were correlated with better 6MWD and with less breathlessness on the mMRC at baseline. However, there was no association between changes in SPPB scores and changes in either 6MWD or mMRC scores during PR.

It is well documented that exercise training improves physical performance in COPD [1].

The changes in SPPB scores following PR observed in the present study are similar to the results found in exercise intervention studies of older adults [22, 23]. The changes correspond with clinically meaningful difference and showed a medium effect size, indicating that the SPPB can be useful for evaluating change in physical performance after interventions in COPD.

As for the SPPB subtests, the 4MGS and 5STS improved significantly, and had a medium and large effect size, respectively [19]. These results are consistent with previous reports [24, 25]. In our study, the 5STS seemed especially responsive to PR. This may be a result of the focus on bilateral leg press at high loads in the exercise program. An increase in leg strength may also explain the improvement in gait speed as strength training can improve gait speed [26]. Patel et al. [9] have previously reported quadriceps strength to be predictive of SPPB scores. As quadriceps muscle dysfunction is common [27] and predicts mortality in COPD [28], it is encouraging to find that PR may improve 4MGS, 5STS, and SPPB scores.

In the present study, SPPB scores were moderately correlated with 6MWD at baseline. However, the mean change in 6MWD were not statistically significant (Table 2) and the changes in SPPB scores were not significantly associated with changes in 6MWD during PR. There are several possible explanations for this result. First, there were missing data in the post-test 6MWD (n = 35 vs. n = 45 at baseline). Second, the follow-up period in this study was only 4 weeks. Early strength gains due to neural adaptations are often present in the beginning of a training period [29], while effects on endurance may take a couple of months [30]. And third, the SPPB may relate more to muscle strength than muscle endurance in patients with COPD, as suggested by Patel et al. [9]. If this is the case, the SPPB may be a useful supplement to the 6MWD, not only because the SPPB is a short and simple test that allows us to measure physical function more often and in different settings than the 6MWD, but also because the SPPB most likely measures a different aspect of physical function compared to the 6MWD (strength vs. endurance).

There was no relationship between SPPB scores and FEV1% at baseline in the present study. This is consistent with previous findings [4, 9]. It is likely that factors other than pulmonary function are important contributors to SPPB scores in patients with COPD.

We found a moderate correlation between SPPB scores and mMRC scores, but there was no correlation between changes in mMRC scores and changes in SPPB scores. Although mMRC scores did improve with PR, improvements were small and of questionable clinical significance. Therefore, the lack of association between changes in SPPB scores and changes in mMRC scores during PR in this study may be a consequence of the mMRC not being very sensitive to change [31].

There was no correlation between SPPB scores and CAT in this study. The SPPB is a short test of lower extremity function and may not reflect the more complex aspects of disease-related problems. However, exercise capacity [32] and the SPPB [33] has been linked to DSQoL in previous studies. Further research may be useful before making conclusions on this subject.

## Further studies

There is a need for larger randomized controlled trials to determine the usefulness of the SPPB in evaluating short- and long-term effects of interventions aimed at improving physical performance in COPD. There is also a need for identifying simple, reliable and valid tools for evaluating physical performance in patients who fall outside the range of the SPPB, for use in settings where the usual field walking tests are unsuitable.

## Conclusion

SPPB scores improved following a four-week in-patient PR program. The SPPB may be a useful tool for evaluating physical performance in patients with COPD before and after pulmonary rehabilitation. However, the test has a substantial ceiling effect affecting high-functioning patients.

## Limitations

- No direct measure of leg strength
- No control-group or blinding
- Small sample size (limited generalization to other populations)
- Missing data led to lack of analysis of changes in CAT scores.
- The SPPB may not capture changes in high functioning patients because of a ceiling effect. For the 12 patients with a SPPB baseline score of 11 points (one point from the maximum score), greater improvements (corresponding to changes greater than one point) is not reflected in the SPPB.
- Some patients with very severe COPD and/or high mMRC dyspnea score performed unexpectedly well on the SPPB. Longer physical performance tests may identify functional limitations due to dyspnea more accurately than the shorter SPPB.

## Abbreviations

CAT: COPD assessment test
COPD: chronic obstructive pulmonary disease
DSQoL: disease-specific quality of life
FEV1%: predicted forced expiratory volume in one second
mMRC: modified Medical Research Council dyspnea scale
SPPB: short physical performance battery
PR: pulmonary rehabilitation
4MGS: four-meter gait speed
5STS: five sit-to-stand
6MWD: six-minute walk distance
6MWT: six-minute walk test
